# Minimal Patient Clinical Variables to Accurately Predict Stress Echocardiography Outcome: Validation Study Using Machine Learning Techniques

**DOI:** 10.2196/16975

**Published:** 2020-05-29

**Authors:** Mohamed Bennasar, Duncan Banks, Blaine A Price, Attila Kardos

**Affiliations:** 1 School of Computing and Comms The Open University Milton Keynes United Kingdom; 2 School of Life, Health and Chemical Sciences The Open University Milton Keynes United Kingdom; 3 Department of Cardiology Milton Keynes University Hospital NHS Foundation Trust Milton Keynes United Kingdom

**Keywords:** stress echocardiography, coronary heart disease, risk factors, machine learning, feature selection, risk prediction

## Abstract

**Background:**

Stress echocardiography is a well-established diagnostic tool for suspected coronary artery disease (CAD). Cardiovascular risk factors are used in the assessment of the probability of CAD. The link between the outcome of stress echocardiography and patients’ variables including risk factors, current medication, and anthropometric variables has not been widely investigated.

**Objective:**

This study aimed to use machine learning to predict significant CAD defined by positive stress echocardiography results in patients with chest pain based on anthropometrics, cardiovascular risk factors, and medication as variables. This could allow clinical prioritization of patients with likely prediction of CAD, thus saving clinician time and improving outcomes.

**Methods:**

A machine learning framework was proposed to automate the prediction of stress echocardiography results. The framework consisted of four stages: feature extraction, preprocessing, feature selection, and classification stage. A mutual information–based feature selection method was used to investigate the amount of information that each feature carried to define the positive outcome of stress echocardiography. Two classification algorithms, support vector machine (SVM) and random forest classifiers, have been deployed. Data from 529 patients were used to train and validate the framework. Patient mean age was 61 (SD 12) years. The data consists of anthropological data and cardiovascular risk factors such as gender, age, weight, family history, diabetes, smoking history, hypertension, hypercholesterolemia, prior diagnosis of CAD, and prescribed medications at the time of the test. There were 82 positive (abnormal) and 447 negative (normal) stress echocardiography results. The framework was evaluated using the whole dataset including cases with prior diagnosis of CAD. Five-fold cross-validation was used to validate the performance of the framework. We also investigated the model in the subset of patients with no prior CAD.

**Results:**

The feature selection methods showed that prior diagnosis of CAD, sex, and prescribed medications such as angiotensin-converting enzyme inhibitor/angiotensin receptor blocker were the features that shared the most information about the outcome of stress echocardiography. SVM classifiers showed the best trade-off between sensitivity and specificity and was achieved with three features. Using only these three features, we achieved an accuracy of 67.63% with sensitivity and specificity 72.87% and 66.67% respectively. However, for patients with no prior diagnosis of CAD, only two features (sex and angiotensin-converting enzyme inhibitor/angiotensin receptor blocker use) were needed to achieve accuracy of 70.32% with sensitivity and specificity at 70.24%.

**Conclusions:**

This study shows that machine learning can predict the outcome of stress echocardiography based on only a few features: patient prior cardiac history, gender, and prescribed medication. Further research recruiting higher number of patients who underwent stress echocardiography could further improve the performance of the proposed algorithm with the potential of facilitating patient selection for early treatment/intervention avoiding unnecessary downstream testing.

## Introduction

Cardiovascular disease (CVD) is the leading cause of death in Western societies [1]. In the United Kingdom, 7.4 million people are living with CVD, which is more than twice the number of people who suffer from cancer and Alzheimer disease. More than 43,000 people under the age of 75 die each year due to CVD costing national health services in the United Kingdom about £9 billion (US $11 billion) [2]. Coronary artery disease (CAD) is the most common form of CVD and may lead to sudden death [3].

Diagnosing CAD early can save lives and reduce risk of myocardial infarction and stroke. Diagnostic procedures are typically performed in specialized cardiac centers to diagnose CAD and risk stratify patients using tests such as a stress echocardiogram. Stress echocardiography is a diagnostic tool to assess the functionality of the heart and blood delivery under stress, such as treadmill or bicycle exercise test or following administration of a drug such as dobutamine. Dobutamine is a pharmacological agent administered intravenously to increase the heart rate in a similar way that would occur during physical exercise. During dobutamine stress echocardiography, incremental doses of dobutamine in 3-minute stages are administered until the termination of the test criteria is achieved. The principle of stress echocardiography is to increase the myocardial oxygen uptake/demand; if the supply is insufficient due to blocked heart arteries, echocardiographic features of this mismatch can be detected by identifying regional wall motion abnormalities in the underperfused heart muscle region during the test. Echocardiographic images are acquired at rest, during the intermediate stage, peak stress, and in recovery. The classical criteria were used as a termination of the test (ie, target heart rate achieved, development of typical chest pain symptoms with or without regional wall motion abnormalities, hemodynamically significant arrhythmias, or development of symptomatic hypotension). Positive or abnormal stress echocardiography is defined as developments of new regional wall motion abnormalities. Wall motion abnormalities were defined as hypokinesia if the wall thickness was maintained and the endocardial excursion was between 5 and 2 mm, akinesia if the wall thickness was reduced and the endocardial excursion was less than 2 mm, and dyskinesia if the wall thickness was reduced and the endocardial excursion was outward moving in systole. Dobutamine stress echocardiography has a sensitivity and specificity of 83% and 86%, respectively [4]. A computer-based algorithm in image analysis and interpretation can play a significant role in the early diagnosis of CAD. Many machine learning–based methods have been devolved for image analysis to aid diagnosis and prognostic monitoring of CAD [5].

Machine learning is a term used to define computer algorithms that can be trained to learn the patterns in training data. These algorithms are then effectively able to make predictions on unseen data. The ability of machine learning techniques to learn from experience without any explicit guidelines for the program or following any predefined rules is making these techniques increasingly popular in many domains [6]. Machine learning in health care has enormous potential in supporting health care practitioners in decision making, enhancing diagnostic accuracy, and reducing health care cost [7]. Machine learning can be used as part of a computer-aided clinician decision support system to assimilate patterns and act as an appropriate source of knowledge.

Several frameworks that employ machine learning for CAD prediction have been proposed [8]. These techniques are used either for predicting the outcome of observations or discovering the hidden pattern and structure in the data not readily recognizable to humans. The data often used for this kind of research include patient anthropometric data, blood test results, and data obtained from various investigation modalities used in the diagnosis of CAD such as electrocardiography, computed tomography angiography, and transthoracic echocardiography [8].

Clinical data have been used to predict coronary events: Voss et al [9] used 10 years of follow-up data from 5159 middle-age men with a 6.3% incidence of coronary events during that period of time. Multilayer perceptron was used to build their model. The study involved 57 clinical and laboratory variables to train the multilayer perceptron. The reported results showed that the area under the curve was 0.89. Gharehchopogh and Khalifelu [10] employed deep learning as a learning algorithm for building a prediction model; the learning algorithm was trained using data from 40 participants that included age, sex, hypertension, and smoking. The reported classification accuracy was 0.85 for heart failure cases.

Another study employed machine learning on clinical and laboratory data of 378,256 patients to predict the first CVD event [11]. The data used consisted of 30 attributes including risk factors, laboratory data, medications, and information about history of CVD and other chronic diseases such as poor mental health, chronic obstructive pulmonary disease, kidney disease, and rheumatoid arthritis. The authors applied four machine learning algorithms: random forest, logistic regression, gradient boosting, and neural networks. The reported results showed that the best performance was achieved by the neural network algorithm with a sensitivity of 67.5% and specificity of 70.7%.

In this pilot study, we aimed to investigate the performance of a machine learning algorithm in predicting the stress echocardiography outcome in patients investigated for suspected CAD. Unlike previous research, we are testing a sophisticated feature selection method to investigate the significance of cardiovascular risk factors, current medication, and anthropometric data in this prediction.

## Methods

### Population and Data Sources

The cohort of patients was derived from the Cardiology Department at Milton Keynes University Hospital in the United Kingdom. Anonymized clinical data had been extracted from patients’ electronic records, predominantly based on the very detailed stress echocardiography reports introduced prospectively by one author (AK), a senior cardiologist, at the time of the development of stress echocardiography services in the hospital. We included all patients (n=563) examined using dobutamine stress echocardiography between 2002 and 2004 with available data. However, we excluded 34 patients who had incomplete clinical data about their risk factors, leaving 529 for this study.

This study used real patient data, which can raise some ethical concerns such as the patient’s permission to use their data and any confidential information that may exposed because of this research. This was resolved by having hospital staff, the direct clinical care provider, anonymize the records before they were sent for analysis. This study was registered by the institutional clinical governance department, Milton Keynes University Hospital (clinical governance project reference number: 33).

Table 1 summarizes patient characteristics for the whole population and separately for the two groups with positive and negative stress echocardiography results. All of these patients had a complete dataset for the anthropometric variables; risk factors such as gender, age, weight, family history (defined as having a first-degree relative who had a myocardial infarction or died suddenly below the age of 60 years), diabetes, smoking status, hypertension, hypercholesterolemia, and prior history of CAD; prescribed medication related to CAD including beta receptor blockers, calcium channel blockers, angiotensin-converting enzyme inhibitor/angiotensin receptor blocker (ACE-I/ARB), antiplatelets, nitrates, statins, and diuretics; and the stress echocardiography results. The features in the table describe the number of patients who have that risk factor positive, for example; 306 of the total 529 participants had hypertension, and 313 of them had abnormal serum cholesterol level.

**Table 1:** Characteristics of patients and their stress echocardiography outcome.

Characteristic			Total (n=529)		SE^a^ positive (n=82)		SE negative (n=447)	
**Risk factor**								
	Sex, male, n (%)		249 (47.1)		61 (74.4)		188 (42.1)	
	Age in years, mean (SD)		61.23 (11.83)		62.92 (10.56)		60.93 (12.06)	
	Weight (kg), mean (SD)		80.82 (17.25)		83.06 (15.88)		80.45 (17.49)	
	Hypertension, n (%)		306 (57.8)		42 (51.2)		264 (59.0)	
	Hypercholesterolemia, n (%)		313 (59.2)		50 (61.0)		263 (58.8)	
	**Smoking, n (%)**							
		Ex-smoker		107 (20.2)		24 (29.3)		83 (18.5)
		Nonsmoker		330 (62.4)		40 (48.8)		290 (64.8)
		Smoker		92 (17.4)		18 (22.0)		74 (16.5)
	Diabetes mellitus, n (%)		99 (18.7)		19 (23.2)		80 (17.8)	
	Family history, n (%)		223 (42.2)		35 (42.7)		188 (42.0)	
	Prior history of CAD^a^, n (%)		123 (23.3)		40 (48.8)		83 (18.5)	
**Medication, n (%)**								
	Beta receptor blocker		281 (53.1)		58 (70.7)		223 (49.8)	
	Calcium channel blocker		137 (25.9)		22 (26.8)		115 (25.7)	
	ACE-I/ARB^a^		258 (48.8)		59 (72.0)		199 (44.5)	
	Antiplatelet therapy		344 (65.0)		64 (78.0)		280 (62.3)	
	Nitrate		159 (30.1)		36 (43.9)		123 (27.5)	
	Statin		314 (59.4)		59 (72.0)		255 (57.0)	
	Diurectic		129 (24.4)		23 (28.0)		106 (23.7)	

^a^SE: stress echocardiography

^a^CAD: coronary artery disease.

^a^ACE-I/ARB: angiotensin-converting enzyme inhibitor/angiotensin-receptor blocker.

### Proposed Framework

**Figure 1:**
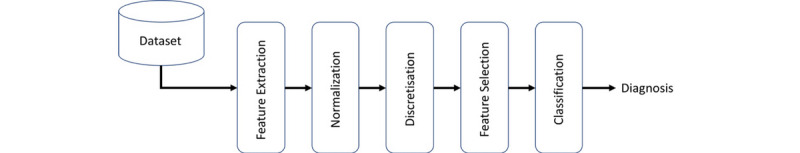
Experiment framework.

The collected data were used to predict the outcome of the stress test based on the patient’s clinical information. Figure 1 shows the architecture of the framework that was used to study the risk factors and medication (referred to as features in this article); we then used these features to investigate the prediction power of this clinical data. Raw data were received as a mixture of text and numerical values. Therefore, the first stage in the proposed framework was the preprocessing stage where natural language processing was used to extract and quantify the needed information from the text, including sex, age, weight, risk factors, medications, and the final outcome of the stress test (positive/negative). The criteria shown in Table 1 were used to convert the text into numerical values.

Feature normalization is the second stage used for continuous features (age and weight), which are normalized using the following equation for normalizing continuous features: 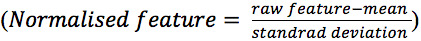.

Two normalized features were then discretized using the equal width discretization method: 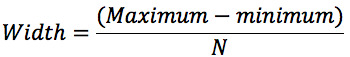, where *N* is the number of bins [12]. In this method, the value of these features is allocated to one of the decimal numbers between 1 and 10. This method divides the range of the feature values into 10 bins of equal width.

Each feature value is assigned to a bin based on the range into which it falls. The reason for the discretization stage is that most of the machine learning algorithms perform better with discretized data [13]. Due the bias of the feature selection stage on the continuous features [14], the discretization stage is also needed to discretize these features before they were submitted to the feature selection stage.

Feature selection, the fourth stage in this framework, is a set of techniques used to measure the significance of each feature for predicting the class label (outcome of the stress test). In this study, the joint mutual information maximization (JMIM) filter feature selection method [15] is used to rank the features according to the amount of information the feature adds to the selected subset. The method measures the amount of information that each feature shares with the class. At the end of this stage, all features (sex, age, weight, risk factors, and medications) will be ranked based on their significance in predicting the class label. This method has been developed based on information theory [16], and the mechanism of the method is explained below.

The value of mutual information between any two variables can be calculated using entropy. It is the amount of uncertainty about a random variable. Suppose *F* = {*f*_1_,*f*_2_,....*f*_N_} is a discrete variable and *C* = {*c*,*c*,....*c*_N_} is a class label; the probability density function is 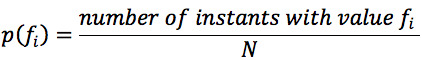.

The mutual information equation between *F* and *C* is as follows:

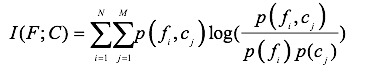



The JMIM method employs the maximum of the minimum criterion. The feature selected by the JMIM method is the one that maximizes the goal function, shown below, where *I*(*f*_i_,*f*_s_;*C*) is the joint mutual information between the candidate feature and the features already selected in the previous iteration. The method employs the forward greedy search algorithm, seen below the equation.

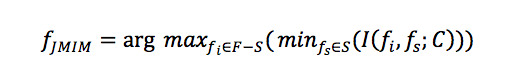

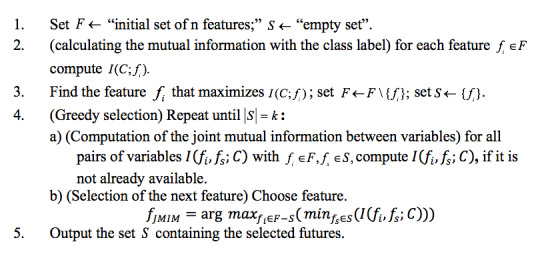





The method does not rank the features based on their individual discriminative power, it selects the features that provide the most information as a subgroup; the interaction information between the features is important in selecting the next significant feature. Therefore, if the list of submitted features is changed, the rank order may be different. The whole dataset was submitted to the JMIM method to identify the significant subset of features (clinical variables). Smialowski et al [17] reported that the feature selection stage should be included within folds-cross validation. However, that can cause instability to the results of the feature selection as submitting data with different instances may lead to different values of probability density function which consequently may lead to changes in the order of the significant features. This paper aims to define the clinical variables that can best predict the outcome of stress echocardiography in the diagnosis of CAD. Therefore, the whole dataset was used at the feature selection stage to take advantage of each valuable instance in the data.

The outcome of the stress test was predicted in the classification stage. Two alternative classifiers were tested at this stage: support vector machine (SVM) [18] and random forest classifiers [19]. The performance of each classifier was evaluated using 5-fold cross-validation. The dataset is imbalanced; there are more than 4 times the negative stress echocardiograms in the data than positive stress echocardiography cases. To overcome this problem, more weight was given to the minority class, and a ratio of 4:1 has been used with SVM; this means giving the minority class 4 times the weight that is given to the majority class. Due to this skewness in the number of classes, classification accuracy will not be a good measure for the performance, as it will be affected mainly by the ability of the classifier to recognize the majority of classes correctly. Therefore, sensitivity and specificity were used to provide a measure for the performance of the classifier in correctly classifying each class.

The data were randomly divided into 5 folds, with 4 of them used to train the classifier and 1 for testing, and then this process was repeated 4 more times, at each time 4 folds were used for training and 4 of the folds that had never been used for testing before was used to test the classifier. At each time, the accuracy, sensitivity, and specificity were calculated. The overall accuracy, sensitivity, and specificity are the average of the 5.

To find the subset of features that produces the best prediction performance, the classifier is trained and tested after adding every feature according to its rank identified at the feature selection stage.

## Results

The proposed framework was used to study the whole dataset including the risk factors and medications, and it was also used to study a subset of the dataset that excluded the cases with prior CAD to investigate the influence of this variable on the performance of the model. Table 2 shows the characteristics of this subset of patients referred to in the rest of this paper as the subdataset.

The prevalence of abnormal stress echocardiography was 15.5%. A total of 447 patients had negative stress echocardiography results (84.5%). There were fewer women than men within the positive group, and the opposite was true with the negative stress echocardiography results. Mean age was 62.92 (SD 10.56) years and 60.93 (SD 12) years in the positive and negative groups, respectively (Table 1).

The feature selection stage was used to rank the features (clinical variables) in the whole dataset, and the significant features for the whole dataset are depicted by Table 3. The table shows that for the whole cohort of patients, CAD is the most significant feature for predicting stress echocardiography outcome, followed by sex, ACE-I/ARB use, and smoking status.

The results showed that prior CAD has the strongest power to distinguish between positive and negative stress echocardiography results. Sex appeared second because most of the positive cases were male, and most of the negative were female. ACE-I/ARB use was the only applied medication among the five most significant features. On the other hand, age, family history, and diabetes appeared the least contributory features in this model.

**Table 2:** Characteristics of patients and their stress echocardiography outcome in those with no prior ischemic heart disease.

Characteristic			SE^a^ positive (n=42)	SE negative (n=364)
**Risk factor**				
	Sex, male, n (%)		33 (78.6)	147 (40.4)
	Age in years, mean (SD)		64.28 (9.80)	60.96 (12.20)
	Weight (kg), mean (SD)		80.31 (13.18)	80.46 (17.37)
	Hypertension, n (%)		25 (59.5)	216 (59.3)
	Hypercholesterolemia, n (%)		33 (78.6)	219 (60.1)
	**Smoking, n (%)**			
		Ex-smoker	13 (31.0)	13 (3.5)
		Nonsmoker	17 (40.5)	227 (62.3)
		Smoker	12 (28.6)	65 (17.8)
	Diabetes mellitus, n (%)		6 (14.3)	67 (18.4)
	Family history, n (%)		17 (40.5)	132 (36.2)
**Medication, n (%)**				
	Beta receptor blocker		28 (66.7)	166 (45.6)
	Calcium channel blocker		9 (21.4)	89 (24.4)
	ACE-I/ARB^a^		32 (76.2)	148 (40.6)
	Antiplatelet therapy		34 (81.0)	216 (59.3)
	Nitrate		24 (57.1)	95 (26.1)
	Statin		29 (69.0)	201 (55.2)
	Diurectic		12 (28.6)	87 (23.9)

^a^SE: stress echocardiography.

^a^ACE-I/ARB: angiotensin-converting enzyme inhibitor/angiotensin receptor blocker.

**Table 3:** Feature rankings.

No	Feature
1	Prior diagnosis of coronary artery disease
2	Sex
3	ACE-I/ARB^a^
4	Weight
5	Smoking status
6	Beta receptor blocker
7	Hypercholesterolemia
8	Antiplatelet therapy
9	Statin
10	Nitrate
11	Hypertension
12	Calcium channel blocker
13	Diuretic
14	Diabetes mellitus
15	Family history
16	Age

^a^ACE-I/ARB: angiotensin-converting enzyme inhibitor/angiotensin-receptor blocker.

Feature selection has been also applied on the subdataset. The order of the features was slightly different as the prior CAD feature was excluded from the data. Table 4 depicts the order of the features, and it shows that sex, ACE-I/ARB, cholesterol, nitrates, and smoking status are the five most significant features. The only difference from the previous results when the whole dataset was used is the swap between serum cholesterol and smoking status. Serum cholesterol status became the third most significant feature followed by nitrates medication, which was not among the most important.

**Table 4:** Feature ranking in the model for patients with no prior ischemic heart disease.

No	Feature
1	Sex
2	ACE-I/ARB^a^
3	Hypercholesterolaemia
4	Nitrate
5	Smoking status
6	Statin
7	Weight
8	Beta receptor blocker
9	Antiplatelet therapy
10	Hypertension
11	Diuretic
12	Calcium channel blocker
13	Diabetes mellitus
14	Family history
15	Age

^a^ACE-I/ARB: angiotensin-converting enzyme inhibitor/angiotensin-receptor blocker.

As mentioned earlier, two classification algorithms were used in this study: SVM and random forest. The results showed that the performances of the two classifiers were close to each other. However, the SVM slightly outperformed the random forest classifier. In this paper, only results produced by the SVM are presented.

**Figure 2:**
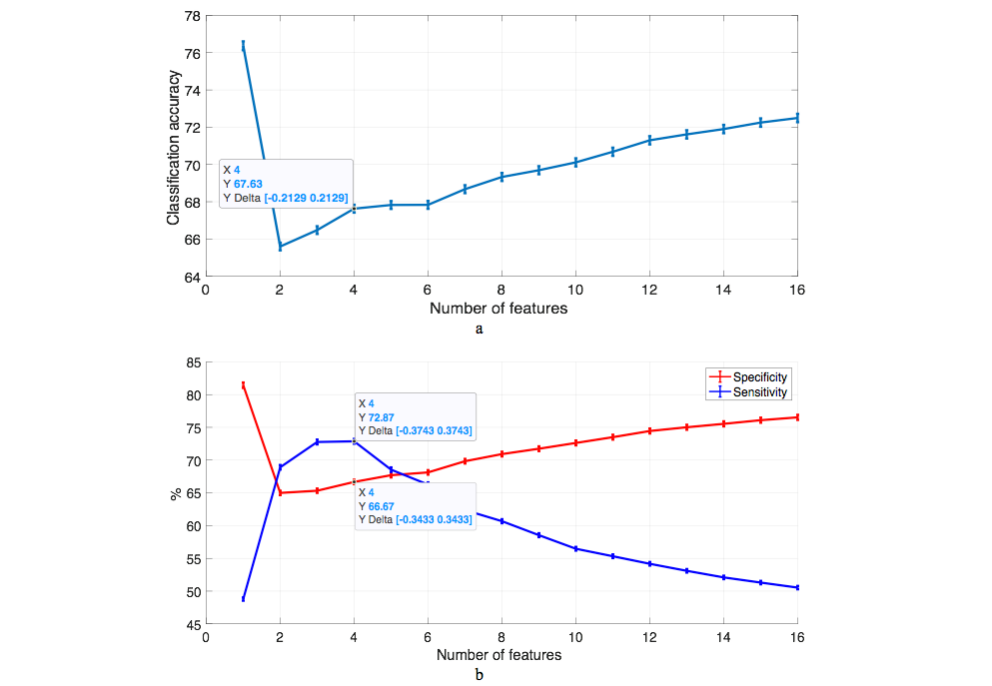
Performance of support vector machine classifier: (a) classification accuracy ± standard error and (b) sensitivity and specificity ± standard error.

Figure 2 shows that the best trade-off between sensitivity and specificity, 72.87% and 66.67%, respectively, was achieved by the subset of the most significant four features (prior CAD, sex, weight, and ACE-I/ARB use). The classification accuracy was 67.63%. The figure also showed that when more features were added, sensitivity started to decrease and specificity started to increase; therefore accuracy is correlated more with sensitivity due to this skewness in the number of classes. This drop in sensitivity means that the rest of the features are either redundant or irrelevant for recognizing positive cases. When whole features were used only about 50% of the positive cases were classified correctly. On the other hand, random forest showed a slightly lower performance when the value of sensitivity, specificity, and classification accuracy were all the same (69.2%); this figure has been achieved with the most significant four features.

**Figure 3:**
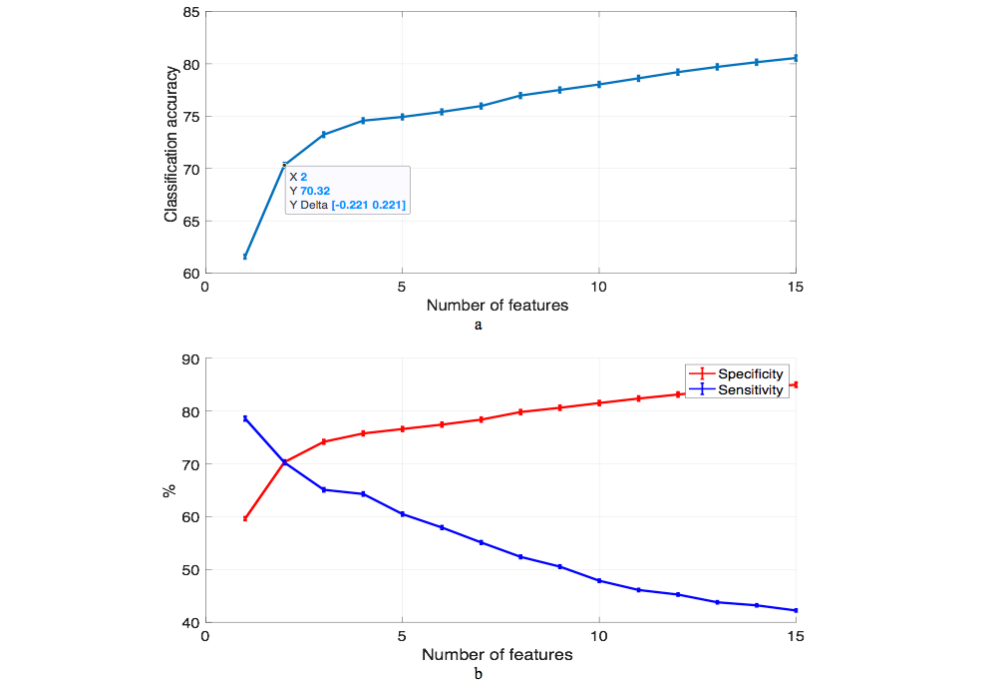
Performance of support vector machine classifier for subdataset: (a) classification accuracy ± standard error and (b) sensitivity and specificity ± standard error.

The experiment was repeated on data from patients with no known prior CAD. The performance of the classification stage is depicted in Figure 3. The sensitivity was slightly affected by excluding patients with CAD as a feature from the data, however, the specificity increased. The classifier produced the best trade-off between sensitivity and specificity, both 70.24%, with only using two features (sex and ACE-I/ARB use). The accuracy also increased to 70.32% due to the increase of the specificity figure.

To test the robustness of the proposed framework, the experiment was repeated again and the model trained without sex features. The results showed that the best performance was achieved with the best four features (prior CAD, ACE-I/ARB, beta receptor blocker, and smoking status): 72.87% and 60.23%, respectively. The accuracy decreased to 62.19%.

## Discussion

### Principal Findings

Feature selection is used as part of the proposed framework; these techniques have the capability to investigate the multidimensional relation between features and class label. In previous research [11], classifiers were used to evaluate the significance of the features based on their importance for the classification algorithm. The selected features are very specific to the classifier. This study employed the classifier independent feature selection method (JMIM) to investigate the relation between features of clinical data and class label (test outcome) in the context of the features that were selected in the previous iterations. This means that once we have selected the features, they can be used with any classifier. SVM and random forest classifiers were tested in this study, and SVM slightly outperformed random forest in our dataset.

The results of the feature selection and classification stages showed that prior CAD is the most important risk factor for distinguishing between positive and negative cases. Sex, weight, and smoking status are among the group of most significant five features, and the only current medication that is within this group is ACE-I/ARB. Hypertension, diabetes, and positive family history are shown as the least significant features for the discrimination task. Only four features were needed to achieve the best performance (prior CAD, sex, weight, and ACE-I/ARB use), which means by knowing only this information about patients, the proposed framework is able to classify 72.87% of the positive cases and 66.67% of the negative cases correctly, outperforming the previous study [11]. ACE-I/ARB is used for several cardiovascular conditions and secondary prevention after an acute coronary event. This feature carries information about these conditions, and that is why it is the most powerful predictor of stress echocardiography outcome. For patients with no prior history of CAD, knowing the sex and whether the patient is taking ACE-I/ARB is sufficient to predict stress echocardiography outcome in the majority of cases.

To study the robustness of the framework, performance was tested without any information about prior diagnosis of CAD. Once this was tested, the order of the feature changed; cholesterol and nitrate medication became among the most significant of the five features. The feature weight was less significant in this model. This change in the order of the features can be attributable to the information interaction between them.

Because prior diagnosis of CAD is such a powerful predictor of a positive stress echocardiogram, the other features contribute so little information by comparison, and it is hard to see their value. However, once these patients are removed from the dataset, we can see the predictive power of the other features for patients with no previous history of CAD.

Features like age, diabetes, and family history are shown to be less significant for discriminating between positive and negative cases. It also showed that information about medications added significant value and could enhance the discrimination power of the clinical data. It also showed that interaction between features is important and can affect the order of the selected subset. Moreover, increasing the granularity of the value of the risk factors may improve their discriminative power by using continuous instead of categorial variables.

### Strengths

To our knowledge, this is the first study that has investigated applying machine learning techniques to a simple dataset of patient anthropometrics, cardiovascular risk factor profiles, and cardioactive medications to predict positive or abnormal stress echocardiography results. The study also investigated the performance of different machine learning techniques and employed a sophisticated feature selection method to study the significance of the clinical attributes. This method considers the interaction between clinical variables when analyzing their significance and the class label. The proposed framework outperforms the other tools that have been proposed in the literature [11] in predicting CAD by more than 9%. The proposed framework can also be employed on data collected using other cardiovascular stress tests aimed to detect inducible ischemia.

### Limitations and Future Work

In this paper, we report preliminary results using only 529 patients. The data includes only anthropometric and clinical data that has been collected during the patient’s hospital visit. Including more data from patient medical records could enhance the generic behavior of any proposed model and improve the performance of the developed model. As we have a large dataset going back nearly 20 years, the model could be extended to predict mortality due to a cardiovascular event.

### Conclusions

Machine learning techniques can offer the very promising prospect of faster and more accurate diagnosis (especially for high-risk groups), prioritizing higher risk patients and increasing the capacity of clinicians. However, it is well known that most machine learning techniques are considered to be black boxes, where the model produces results that are difficult to interpret. Despite the black box nature of various machine learning approaches, feature selection techniques can improve understanding of the relationship between the diagnosis and clinical attributes. Data visualization methods can improve understanding of the produced model and interpretation of the output.

None of the clinical information detailing the results of the positive stress test such as wall motion score index were included with the clinical data. Inclusion may further differentiate between high- and low-risk patients.

## Abbreviations

ACE-I/ARB: angiotensin-converting enzyme inhibitor/angiotensin-receptor blocker
CAD: coronary artery disease
CVD: cardiovascular disease
JMIM: joint mutual information maximization
SVM: support vector machine
